# Application of AMR in evaluating microvascular dysfunction after ST‐elevation myocardial infarction

**DOI:** 10.1002/clc.24196

**Published:** 2023-11-24

**Authors:** Hao Wang, Qi Wu, Lang Yang, Long Chen, Wen‐Zhong Liu, Jun Guo, Jing‐Song Xu

**Affiliations:** ^1^ Department of Cardiology The Second Affiliated Hospital of Nanchang University Nanchang China; ^2^ The First Affiliated Hospital of Nanchang University Nanchang China; ^3^ Shanghai Pulse Medical Technology Inc. Shanghai China

**Keywords:** angiography‐derived microcirculatory resistance (AMR), coronary microvascular dysfunction (CMD), major adverse cardiovascular events (MACE), quantitative flow ratio (QFR), ST‐elevation myocardial infarction (STEMI)

## Abstract

**Background:**

A guidewire‐free angiography‐derived microcirculatory resistance (AMR) derived from Quantitative flow ratio (QFR) exhibits good diagnostic accuracy for assessing coronary microvascular dysfunction (CMD), but there are no relevant studies supporting the specific application of AMR in patients with ST‐elevation myocardial infarction (STEMI). The study aims to evaluate CMD in patients with STEMI using the AMR index.

**Methods:**

This study included patients with STEMI who underwent percutaneous coronary intervention (PCI) from June 1, 2020 to September 28, 2021. All patients were divided into two groups: the CMD (*n* = 215) and non‐CMD (*n* = 291) groups. After matching, there were 382 patients in both groups.1‐year follow‐up major adverse cardiac events (MACEs) were evaluated.

**Results:**

After matching, the primary endpoint was achieved in 41 patients (10.7%), with 27 and 14 patients in the CMD and non‐CMD groups, respectively (HR 1.954 [95% CI 1.025–3.726]; 14.1% versus 7.3%, *p* = .042). Subgroup analysis revealed that 18 patients (4.7%) were readmitted for heart failure, with 15 and 3 in the CMD and non‐CMD groups, respectively (HR 5.082 [95% CI 1.471–17.554]; 7.9% versus 1.6%, *p* = .010). Post‐PCI AMR ≥ 250 was significantly associated with a higher risk of the primary endpoint and was its independent predictor (HR 2.265 [95% CI 1.136–4.515], *p* = .020).

**Conclusion:**

The retrospective use of AMR with a cutoff value of ≥250 after PCI in patients with STEMI can predict a significant difference in the 1‐year MACE rates when compared with a propensity score‐matched group with normal AMR.

## Graphical abstract

A guidewire‐free and adenosine‐free angiography‐derived microcirculatory resistance (AMR) derived from quantitative flow ratio with flow velocity calculation exhibits good diagnostic accuracy for assessing coronary microvascular dysfunction. In our study, post‐PCI AMR ≥ 250 mmHg × s/m was significantly associated with a higher risk of the primary endpoint and was its independent predictor.

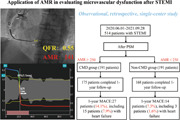

## 1 INTRODUCTION

Coronary microvascular dysfunction (CMD) is a significant factor associated with myocardial ischemia development in various cardiovascular diseases, including coronary artery disease (CAD).^1^, ^2^ Notably, postreperfusion CMD is often observed in patients with ST‐elevation myocardial infarction (STEMI), with the presence of microvascular obstruction (MVO) being associated with unfavorable clinical outcomes.^3^

CMD is quantitatively and repeatedly evaluated using the index of microcirculatory resistance (IMR) based on intracoronary pressure and temperature changes, which can be measured using a pressure guidewire.^4^ However, the clinical application of IMR remains limited owing to the requirement of dilating drugs, pressure guidewires, long measurement times, and high costs. In patients with CAD, functional assessment is gaining importance. Quantitative flow ratio (QFR) is an emerging angiography‐based assessment method that helps calculate fractional flow reserve values via three‐dimensional coronary reconstruction and fluid dynamics.^5^ Several large‐scale randomized controlled trials, including the FAVOR Pilot and FAVOR II studies, have confirmed the accuracy of QFR, further validating the diagnostic value of QFR and accuracy in assessing coronary ischemia.^5^, ^6^, ^7^, ^8^, ^9^ Furthermore, in the FAVOR III study, researchers compared visual assessment and QFR assessment and observed that myocardial infarction (MI) and ischemia‐driven revascularization are less frequent in the QFR‐guided group than in the angiography‐guided group.^10^ Indeed, QFR eliminates the requirement of pressure guidewires and is a suitable strategy for assessing coronary ischemia.^6^ Moreover, a guidewire‐free and adenosine‐free angiography‐derived microcirculatory resistance (AMR) derived from QFR with flow velocity calculation exhibits good diagnostic accuracy for assessing CMD; therefore, it can be an effective clinical alternative to invasive pressure guidewire‐based IMR.^11^

Although QFR is an important method to functionally assess patients with CAD, there are no relevant studies supporting the specific application and assessment value of AMR in patients with STEMI. Therefore, in the present study, we elucidated the value of AMR in assessing the incidence of major adverse cardiac events (MACEs) by evaluating postoperative AMR and the follow‐up characteristics of patients with STEMI.

## 2 MATERIALS AND METHODS

### 2.1 Study design

This single‐center observational study included patients with STEMI who successfully underwent percutaneous coronary intervention (PCI) at The Second Affiliated Hospital of Nanchang University from June 1, 2020 to September 28, 2021. The microcirculatory status of the culprit vessel in all enrolled patients was noninvasively evaluated using AMR. Postoperative AMR, QFR, and 1‐year follow‐up data were collected.

Based on a postoperative AMR value of 250 mmHg × s/m (owing to the absence of an established AMR cutoff value for CMD, relevant data from a recent study were used^11^), all patients were divided into two groups: the CMD (AMR ≥ 250, *n* = 215) and non‐CMD (AMR < 250, *n* = 291) groups. After matching, there were 382 patients in both groups.

### 2.2 Patient population

Adult patients with STEMI who underwent PCI within 12 h of symptom onset had ≥50% stenosis of the lesion diameter on initial angiography. In patients with STEMI who were undergoing emergency PCI, the culprit vessel was physiologically assessed after thrombus aspiration, balloon dilatation flow restoration, and/or primary PCI completion. STEMI was defined as the occurrence of persistent chest pain for at least 30 min, with ST elevation of >2 mm in at least two contiguous leads or a new left bundle branch block.^12^ The culprit vessel was identified based on the following aspects^1^: angiographic presentation matching the presence of plaque instability or thrombus and^2^ electrocardiographic and echocardiographic findings. Two angiographic images with a projection angle separation of at least 25° were obtained. Then, using the local site network for QFR and AMR calculation, the data were transferred to the AngioPlus system (Pulse Medical Imaging Technology, Shanghai, China). Patients were excluded if QFR or AMR could not be calculated because of^1^ the poor quality of angiographic images and^2^ the presence of severe vascular curvature and overlap.^7^, ^13^

The retrospective study was approved by the Institutional Review Boards at The Second Affiliated Hospital of Nanchang University (approval no. 2022‐07) and was conducted in accordance with the 1964 Helsinki Declaration and its later amendments or comparable ethical standards. Informed consent was waived by our Institutional Review Board because of the retrospective nature of our study.

### 2.3 Data collection

Using medical records, the following parameters were retrospectively collected: age, sex, cigarette smoking, AMI or PCI history, and clinical comorbidities, including hypertension, diabetes, and hyperlipidemia. Serum biochemical markers, including glucose, low‐density lipoprotein‐cholesterol (LDL‐c), brain natriuretic peptide (BNP), C‐reactive protein, creatinine, and troponin I, were measured at the clinical laboratory of the hospital using routine automated techniques.

### 2.4 QFR and AMR analyses

The AngioPlus system (Pulse Medical Imaging Technology) was used according to standard operating procedures to calculate AMR and QFR. Before angiographic assessment, an angiographic view table was provided to the operators. Analysis was performed by an experienced analyst who was blinded to the follow‐up data. The detailed method for single‐view QFR and AMR computation has been reported in previous studies.^11^, ^14^ Briefly, the contrast medium was manually injected with a forceful and stable injection or by a pump at a rate of approximately 4 mL/s. As soon as the optimal angiographic view with minimal vessel overlap was chosen, the lumen contours of the coronary arteries were automatically measured; on the other hand, contrast flow velocity was calculated by dividing the length of the vessel centerline with the contrast filling time and then converted into hyperemic flow velocity (Velocity_hyp_).^15^ Subsequently, the analysis frame should have good contrast fill‐in and complete exposure of the lumen contour, with automatic delineation of the lumen boundaries of both the studied vessel and major side branches. Based on the Murray bifurcation fractal law, the reference vessel diameter was reconstructed, considering the step‐down phenomenon across bifurcations.^13^ Finally, based on the fluid dynamic equations with the abovementioned hyperemic flow as the boundary condition, the pressure drop was calculated.^14^ Using the determined pressure drop, the distal coronary pressure (Pd) was obtained. QFR was calculated by dividing Pd by the mean aortic pressure; in contrast, AMR was calculated by dividing Pd with Velocity_hyp_.^11^ Revascularization was used to determine the anatomical information of the target vessel, including lumen diameter and lesion length. The reference vessel was constructed using the system on a healthy segment, which was ideally located proximal and distal to the target lesion.

### 2.5 Clinical follow‐up

The relevant clinical data and 1‐year MACEs of all enrolled individuals were recorded. MACE was defined as a composite of death from any cause, any MI, readmission for heart failure, or any ischemia‐driven revascularization. Telephonic follow‐up or medical record review was used to determine the occurrence of MACE within 1 year.

Cardiac‐related death was defined as death owing to MI, severe arrhythmia, refractory heart failure, or cardiogenic shock. Readmission for heart failure was defined as hospitalization owing to new or worsening signs and symptoms of heart failure, with concurrent noninvasive imaging findings or increased BNP levels and discharge with a diagnosis of congestive heart failure. Spontaneous MI was defined as an increase in creatine kinase or troponin levels above the upper limit of normal, with symptoms of ischemia or ECG findings suggestive of ischemia.^16^ Ischemia‐driven target revascularization was defined as revascularization with at least one of the following^1^: recurrence of angina pectoris^2^; positive noninvasive test result; and^3^ positive invasive physiological test result.

### 2.6 Statistical analysis

Continuous variables were recorded as mean ± standard deviation or median, whereas categorical variables were recorded as counts (percentages). The Kolmogorov–Smirnov test or Shapiro–Wilk test was performed to determine the normality of the data. Pearson's *χ* ^2^ test or Fisher's exact test (as appropriate) was performed to compare categorical variables. The baseline characteristics were balanced using propensity score matching (PSM). For PSM, the 1:1 nearest neighbor matching method was used without replacement and with a caliper of 0.02. The Kaplan–Meier method was used to determine the time‐to‐first event rate of each group. The findings were compared using the log‐rank test. A Cox proportional hazards model with hazard ratios (HRs) and 95% confidence intervals (CIs) was used to determine between‐group differences. Clinically relevant covariates or univariate variables associated with outcomes (*p* < .10) were entered into the multivariate Cox model. The covariates included age, male sex, body mass index, hypertension, diabetes, hyperlipidemia, stroke history, hemoglobin A1C (HbA1c), BNP, albumin, troponin I detection peak, random blood glucose levels, left ventricular ejection fraction (LVEF), and AMR ≥ 250. A *p* Value of <.05 was considered statistically significant; all comparisons were two‐sided. SPSS 26.0 was used to perform all statistical analyses.

## 3 RESULTS

### 3.1 Baseline characteristics

**Figure 1:**
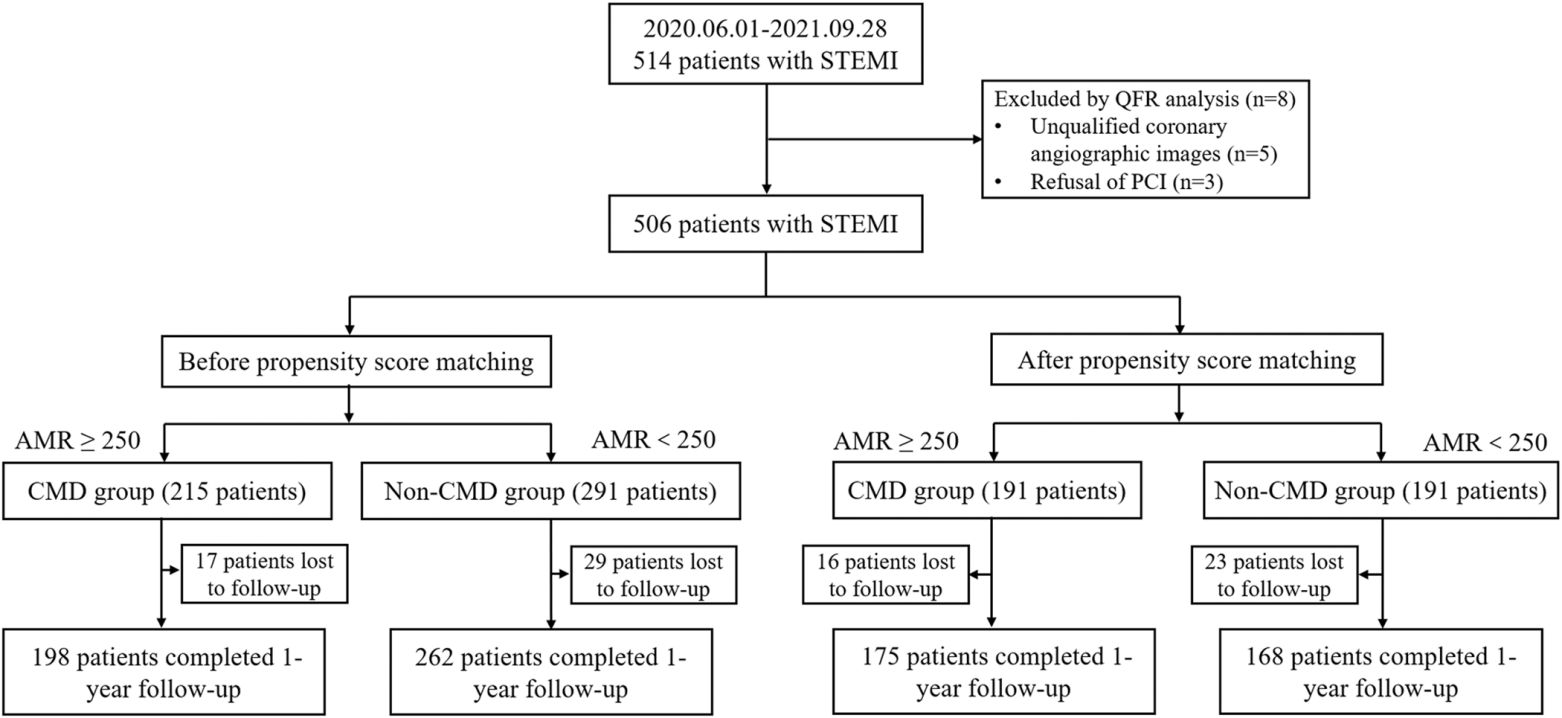
Study flowchart diagram AMR, angio‐derived microcirculatory resistance; CMD, coronary microvascular dysfunction; PCI, percutaneous coronary intervention; QFR, quantitative flow ratio; STEMI, ST‐segment elevation myocardial infarction.

Figure 1 shows that 514 patients with AMI were included in this study; of them, three refused to undergo PCI and five had poor‐quality QFR images; as a result, 506 patients with STEMI were finally included. These patients were divided into two groups: the CMD (AMR ≥ 250, *n* = 215) and non‐CMD (AMR < 250, *n* = 291) groups. Table S1 summarizes the clinical, laboratory, and angiographic characteristics of the patients. The mean age was 63 years, with 416 men (82.2%) and 90 women (17.8%). Furthermore, 137 (27.1%) had diabetes mellitus, 265 (52.4%) had hypertension, and 138 (27.3%) had hyperlipidemia. No significant differences were observed between the CMD and non‐CMD groups in terms of the incidence of hypertension, diabetes mellitus, smoking history, family history of coronary heart disease, stroke history, MI history, antiplatelet drug use, statin use, and LVEF (*p* > .05). Compared with patients without CMD, those with CMD were significantly older [67.00 (56.00–74.00) versus 60.00 (51.75–72.00) years, *p* = .000], had lesser men [167 (77.7%) versus 249 (85.6%), *p* = .022], had a lower body mass index (22.64 [20.28–23.66] versus 22.64 [20.28–25.35] kg/m^2^, *p* = .017), had a lower incidence of hyperlipidemia (47 [21.9%] versus 91 [31.3%], *p* = .019), and lesser use of angiotensin‐converting enzyme inhibitors/angiotensin II receptor blockers (120 [55.8%] versus 192 [66%], *p* = .020). In terms of biochemical indicators, patients with CMD had lower serum albumin levels (37.99 ± 3.97 versus 39.01 ± 3.90 g/L, *p* = .004), higher neutrophil counts (8.09 [5.79–10.46] versus 7.16 [5.45–9.68] × 10^9^/L, *p* = .034), lower lymphocyte counts (1.41 [0.95–1.94] versus 1.59 [1.04–2.37] × 10^9^/L, *p* = .024), higher BNP levels (189.19 [78.09–485.36] versus 142.55 [50–401.82] pg/mL, *p* = .011], and lower LDL‐c levels (2.67 [2.14–3.31] versus 2.85 [2.36–3.44] mmol/L, *p* = .022) than those without CMD. No significant differences were observed in the other indexes between both groups (*p* > .05).

Using PSM with a caliper value of 0.02, 191 pairs were successfully matched between both groups. The covariates that were not balanced before matching were balanced between both groups after matching (*p* > .05, Table S1).

### 3.2 Coronary angiography characteristics

Compared with the non‐CMD group, the incidence of postoperative TIMI blood flow grade 3 was lower in the CMD group [prematch 206 (95.8%) versus 291 (100%), *p* = .000; prematch 184 (96.3%) versus 375 (98.2%), *p* = .015]. Nevertheless, no differences were observed in terms of prematch and postmatch radial artery routing, bivalirudin use, thrombus aspiration, multivessel disease, balloon predilation, balloon postdilation, culprit vessel, number of stents, use of drug‐eluting stents, drug‐eluting balloons, and nondrug balloons between both groups (*p* > .05, Table S2).

### 3.3 Results of AMR and QFR analyses

Table 1 and Figure S1 summarize the data for AMR and QFR analyses. Post‐PCI QFR was significantly higher in the CMD group than in the non‐CMD group [prematch 0.97 (0.95–0.99) versus 0.93 (0.89–0.97), *p* = .000; postmatch 0.97 (0.95–0.99) versus 0.92 (0.87–0.96), *p* = .000]. In contrast, post‐PCI flow velocity was significantly lower in the CMD group than in the non‐CMD group (prematch 13.05 [11.10–14.60] versus 19.8 [17.80–23.40] cm/s, *p* = .000; postmatch 13.10 [11.10–14.60] versus 21.4 [18.60–25.00] cm/s, *p* = .000). However, before and after matching, no statistical differences were observed between both groups in terms of reference vessel diameter, minimum lumen diameter, stenosis diameter, lesion length, total stent length/vessel, in‐stent reference vessel diameter, in‐stent minimum lumen diameter, and in‐stent stenosis diameter (*p* > .05, Table 1).

**Table 1:** Procedural characteristics.

	Before propensity score matching					After propensity score matching				
	All subjects (*n* = 506)	AMR ≥ 250 (*n* = 215)	AMR＜250 (*n* = 291)	Z	*p* Value	All subjects (*n* = 382)	AMR ≥ 250 (*n* = 191)	AMR＜250 (*n* = 191)	Z	*p* Value
Reference vessel diameter, mm	2.85 (2.45‐3.16)	2.85 (2.49‐3.20)	2.85 (2.45‐3.16)	−0.376	0.707	2.85 (2.45‐3.15)	2.85 (2.50‐3.20)	2.80 (2.40‐3.15)	−1.215	0.224
Minimal lumen diameter, mm	0.89 (0.70‐1.00)	0.80 (0.70‐1.00)	0.89 (0.70‐1.00)	−1.432	0.152	0.89 (0.70‐1.00)	0.80 (0.70‐1.00)	0.89 (0.70‐1.00)	−0.593	0.553
Diameter stenosis, %	68.00 (61.00‐74.25)	69.00 (62.00‐74.00)	68.00 (61.00‐75.00)	−0.880	0.379	69.00 (61.00‐75.00)	69.00 (62.00‐75.00)	68.00 (61.00‐75.00)	−0.598	0.550
Lesion length, mm	31.40 (21.28‐40.43)	32.80 (21.70‐40.60)	29.90 (20.80‐39.00)	−1.100	0.271	32.00 (21.68‐40.80)	32.80 (21.70‐40.60)	30.80 (21.60‐41.40)	−0.338	0.735
Total stent length per vessel, mm	33.25 (27.98‐45.90)	36.00 (29.00‐45.33)	33.00 (25.78‐46.00)	−1.537	0.124	35.00 (28.00‐46.00)	36.00 (29.00‐44.00)	33.00 (26.00‐49.50)	−0.940	0.347
In‐stent reference vessel diameter, mm	3.20 (2.85‐3.55)	3.15 (2.84‐3.50)	3.20 (2.89‐3.60)	−1.546	0.122	3.15 (2.85‐3.50)	3.20 (2.85‐3.50)	3.15 (2.85‐3.45)	−0.225	0.822
In‐stent minimal lumen diameter, mm	2.70 (2.40‐3.00)	2.70 (2.40‐3.00)	2.70 (2.40‐3.10)	−1.463	0.144	2.70 (2.30‐3.00)	2.70 (2.30‐3.00)	2.70 (2.30‐3.00)	−0.220	0.826
In‐stent diameter stenosis, %	11.00 (5.00‐18.00)	11.00 (5.00‐18.00)	12.00 (5.00‐19.25)	−0.781	0.435	12.00 (5.00‐18.00)	11.00 (5.00‐18.00)	12.00 (6.00‐19.00)	−1.117	0.264
Pre‐PCI QFR	0.38 (0.19‐0.59)	0.39 (0.21‐0.62)	0.35 (0.18‐0.57)	−1.489	0.137	0.38 (0.18‐0.59)	0.39 (0.19‐0.62)	0.34 (0.17‐0.55)	−1.497	0.134
Post‐PCI QFR	0.95 (0.91‐0.98)	0.97 (0.95‐0.99)	0.93 (0.89‐0.97)	−8.532	0.000*	0.95 (0.91‐0.98)	0.97 (0.95‐0.99)	0.92 (0.87‐0.96)	−8.780	0.000*
Post‐PCI AMR, mmHg*s/m	241.00 (206.00‐280.00)	287.50 (269.00‐315.50)	212.00 (190.00‐235.00)	−19.2 41	0.000*	249.50 (198.00‐288.00)	288.00 (269.00‐315.00)	198.00 (183.00‐222.00)	−16.9 05	0.000*
Post‐PCI Flow velocity, cm/s	16.95 (13.60‐20.80)	13.05 (11.10‐14.60)	19.8 (17.80‐23.40)	−18.5 56	0.000*	16.40 (13.08‐21.40)	13.10 (11.10‐14.60)	21.4 (18.60‐25.00)	−16.4 13	0.000*

*Note*: Values are presented as the mean ± standard deviation, median (25th percentile, 75th percentile) or *n* (%). Asterisk(*) denote statistically significant differences between the two groups (*p*‐value < 0.05).

Abbreviations: AMR, angio‐derived microcirculatory resistance; PCI, percutaneous coronary intervention; QFR, quantitative flow ratio.

### 3.4 1‐year follow‐up characteristics

**Figure 2:**
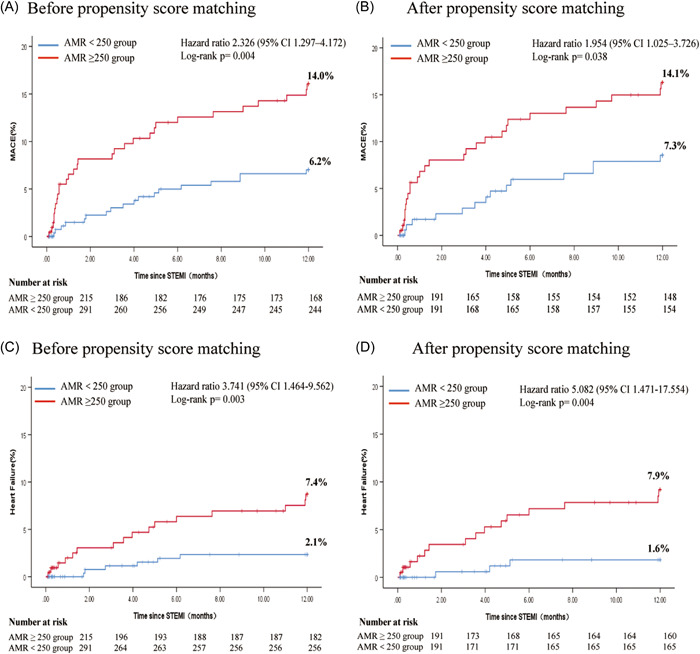
Kaplan−Meier curves for the MACE and heart failure. Comparison of 1‐year MACE rates between patients with AMR ≥ 250 and AMR＜250 group, classified according to the value of AMR (250 mmHg*s/m) is presented. Patients in the AMR ≥ 250 group showed a higher MACE rate and readmission rate of HF than those in AMR＜250 group before PSM (A, C) and after PSM (B, D). AMR, angiography‐derived microcirculatory resistance; MACE, major adverse cardiac events.

Table 2 summarizes the clinical characteristics of both groups at 1‐year follow‐up. The primary endpoint was achieved in 48 patients (9.5%), with 30 and 18 patients in the CMD and non‐CMD groups, respectively (HR 2.326 [95% CI 1.297–4.172]; 14.0% versus 6.2%, *p* = .005). Subgroup analysis revealed that 22 patients (4.3%) were readmitted for heart failure, with 16 and 6 patients in the CMD and non‐CMD groups, respectively (HR 3.741 [95% CI 1.464–9.562]; 7.4% versus 2.1%, *p* = .006). The Kaplan–Meier curves revealed the cumulative incidence of MACE and heart failure readmission (Figure 2). In total, 24 patients (4.8%) had all‐cause death, 2 (0.4%) had MI, and 0 (0%) had target vessel revascularization; no statistical difference was observed between both groups (*p* > .05). Furthermore, during secondary endpoint analysis, 11 (2.2%) cases of cardiac‐related deaths and 13 (2.6%) cases of Noncardiac‐related death were observed; nevertheless, no statistical difference was observed between both groups in terms of cardiac‐ and Noncardiac‐related deaths (*p* > .05).

**Table 2:** 1‐year clinical outcomes.

	Before propensity score matching					After propensity score matching				
	All subjects (*n* = 506)	AMR ≥ 250 (*n* = 215)	AMR＜250 (*n* = 291)	Hazard ratio (95% CI)	*p* Value	All subjects (n = 382)	AMR ≥ 250 (*n* = 191)	AMR＜250 (*n* = 191)	Hazard ratio (95% CI)	*p* Value
Primary endpoint										
MACE, *n* (%)	48 (9.5)	30 (14.0)	18 (6.2)	2.326 (1.297‐4.172)	0.005*	41 (10.7)	27 (14.1)	14 (7.3)	1.954 (1.025‐3.726)	0.042*
Death from any cause, *n* (%)	24 (4.7)	14 (6.5)	10 (3.4)	1.943 (0.863‐4.374)	0.109	21 (5.5)	12 (6.3)	9 (4.7)	1.347 (0.567‐3.196)	0.500
Myocardial infarction, *n* (%)	2 (0.4)	0 (0)	2 (0.7)	0.021 (0.000‐2273.362)	0.512	2 (0.5)	0 (0)	2 (1.0)	0.016 (0.000‐1349.348)	0.473
Ischemia‐driven revascularization, *n* (%)	0 (0)	0 (0)	0 (0)	‐	‐	0 (0)	0 (0)	0 (0)	‐	‐
Readmission for heart failure, *n* (%)	22 (4.3)	16 (7.4)	6 (2.1)	3.741 (1.464‐9.562)	0.006*	18 (4.7)	15 (7.9)	3 (1.6)	5.082 (1.471‐17.554)	0.010*
Major secondary endpoint										
Cardiac‐related death, *n* (%)	11 (2.2)	6 (2.8)	5 (1.7)	1.656 (0.505‐5.426)	0.405	8 (2.1)	4 (2.1)	4 (2.1)	1.004 (0.251‐4.013)	0.996
Noncardiac‐related death, *n* (%)	13 (2.6)	8 (3.7)	5 (1.7)	2.236 (0.731‐6.834)	0.158	13 (3.4)	8 (4.2)	5 (2.6)	1.622 (0.531‐4.958)	0.396

*Note*: Values are *n* (%). Asterisk(*) denote statistically significant differences between the two groups (*p*‐value < 0.05).

Abbrevitions: CI, confidence interval; MACE, Major adverse cardiovascular events.

After PSM, at 1‐year follow‐up, the primary endpoint was achieved in 41 patients (10.7%), with 27 and 14 patients in the CMD and non‐CMD groups, respectively (HR 1.954 [95% CI 1.025–3.726]; 14.1% versus 7.3%, *p* = .042). Subgroup analysis revealed that 18 patients (4.7%) were readmitted for heart failure, with 15 and 3 in the CMD and non‐CMD groups, respectively (HR 5.082 [95% CI 1.471–17.554]; 7.9% versus 1.6%, *p* = .010). In total, 21 (5.5%) had all‐cause death, 2 (0.4%) had MI, and 0 (0%) had target vessel revascularization, with no statistical difference between both groups (*p* > .05). Secondary endpoint analysis revealed that 8 patients (2.1%) had cardiac‐related deaths and 13 (3.4%) had noncardiac‐related deaths, with no statistical difference between both groups (*p* > .05). The higher incidence of heart failure readmission in the CMD group compared with the non‐CMD group was the main composite outcome.

Table 3 summarizes the univariate and multifactor predictors of the primary endpoint. The 14 independent variables of significance were as follows: post‐PCI AMR ≥ 250, age, sex, BMI, diabetes, hypertension, hyperlipidemia, stroke, HbA1c, BNP, albumin, troponin I detection peak, random blood glucose levels, and LVEF. They were included in multifactorial Cox regression analysis using a univariate Cox regression model. Post‐PCI AMR ≥ 250 was significantly associated with a higher risk of the primary endpoint and was its independent predictor (prematched adjusted HR 2.037 [95% CI 1.068–3.888], *p* = .031; postmatched adjusted HR 2.265 [95% CI 1.136–4.515], *p* = .020), in addition to age, BNP, and troponin I detection peak (Table 3).

**Table 3:** Univariate and multivariate analysis results for predictors of 1‐year MACE.

	Before propensity score matching				After propensity score matching			
	Univariable analysis		Multivariable analysis		Univariable analysis		Multivariable analysis	
	HR (95% CI)	*p* Value	HR (95% CI)	*p* Value	HR (95% CI)	*p* Value	HR (95% CI)	*p* Value
AMR ≥ 250	2.326 (1.297‐4.172)	0.005*	2.037 (1.068‐3.888)	0.031*	1.954 (1.025‐3.726)	0.042*	2.265 (1.136‐4.515)	0.020*
Age	1.072 (1.046‐1.099)	0.000*	1.045 (1.015‐1.076)	0.003*	1.078 (1.046‐1.111)	0.000*	1.062 (1.025‐1.101)	0.001*
Male	1.596 (0.830‐3.068)	0.161			1.030 (0.476‐2.230)	0.940		
Body mass index	0.884 (0.801‐0.975)	0.014*	1.002 (0.901‐1.114)	0.972	0.898 (0.807‐0.999)	0.048*	1.010 (0.901‐1.131)	0.868
Diabetes mellitus	0.308 (0.122‐0.778)	0.013*	1.119 (0.298‐4.205)	0.868	0.453 (0.178‐1.154)	0.097	1.786 (0.453‐7.042)	0.407
Hypertension	1.431 (0.802‐2.551)	0.225			1.227 (0.662‐2.274)	0.515		
Hyperlipidemia	0.420 (0.188‐0.936)	0.034	0.692 (0.302‐1.586)	0.384	0.600 (0.266‐1.353)	0.218		
Previous stroke	3.248 (1.656‐6.369)	0.001*	1.703 (0.774‐3.750)	0.186	3.233 (1.584‐6.597)	0.001*	1.735 (0.763‐3.948)	0.189
HBA1C	0.679 (0.498‐0.927)	0.015*	0.936 (0.590‐1.484)	0.778	0.737 (0.539‐1.008)	0.056	0.928 (0.586‐1.468)	0.748
BNP	1.000 (1.000‐1.001)	0.000*	1.000 (1.000‐1.000)	0.014*	1.000 (1.000‐1.001)	0.000*	1.000 (1.000‐1.000)	0.207
Albumin	0.871 (0.813‐0.933)	0.000*	0.958 (0.879‐1.043)	0.322	0.872 (0.807‐0.942)	0.000*	0.954 (0.871‐1.044)	0.305
Troponin I detection peak	1.017 (0.999‐1.034)	0.065	1.023 (1.004‐1.042)	0.016*	1.016 (0.997‐1.037)	0.100	1.024 (1.003‐1.046)	0.024*
Glucose	0.828 (0.716‐0.958)	0.011*	0.856 (0.704‐1.040)	0.118	0.851 (0.735‐0.986)	0.031*	0.818 (0.659‐1.016)	0.069
LVEF	0.947 (0.919‐0.975)	0.000*	0.974 (0.942‐1.006)	0.113	0.946 (0.916‐0.976)	0.000*	0.974 (0.940‐1.009)	0.137

*Note*: Asterisk(*) denote statistically significant differences (*p*‐value < 0.05).

Abbreviations: CI, confidence interval; HR, hazard ratio; other abbreviations as in Table 1.

## 4 DISCUSSION

The currently available diagnostic methods for evaluating CMD include noninvasive tests such as positron emission tomography (PET), cardiac echocardiography, cardiac computed tomography, and cardiac magnetic resonance (CMR) and invasive tests such as coronary angiography, Doppler flow map, coronary flow reserve, and IMR. At present, PET is considered the gold standard reference for noninvasively evaluating CMD; however, the high cost, inability to perform repeated measures, and radiation exposure of PET have limited its use in clinical settings.^17^ Previous studies have frequently elucidated invasive IMR, and evidence suggests that pressure wire‐based IMR can be used to assess postischemic CMD.^18^, ^19^ Fearon et al.^20^ have reported that a high IMR of >40 measured at initial PCI in patients with STEMI predicted long‐term clinical outcomes, including death in heart failure and rehospitalization. Furthermore, the immediate measurement of the IMR of the culprit vessel after successful initial PCI in patients with STEMI can help assess patient prognosis. However, additional requirements of pressure–temperature transducer wires limit the use of IMR in routine procedures.

With advances in functional evaluation approaches, we are no longer limited to using assessment tools such as pressure wire‐based IMR. For the first time, Tebaldi et al.^21^ validated the formula for calculating microvascular resistance based on cQFR data without using pressure guidewires and drug‐induced congestion. Simultaneously, Sheng et al.^22^ reported that QFR calculation may be a useful tool for predicting CMD after STEMI. With advances in research, a study has demonstrated the feasibility of assessing coronary microcirculatory resistance without using intracoronary pressure guidewires and adenosine.^23^ Furthermore, a study published during the same period have reported that the non‐hyperaemic angiography‐derived IMR is prognostically equivalent to invasively measured IMR and may be its viable alternative in patients with STEMI.^24^ In addition, recent studies have reported that the AMR index obtained from a single angiographic view only is a feasible computational alternative to pressure‐guided IMR, with good diagnostic accuracy for assessing CMD.^11^ In patients with AMI, current noninvasive examinations and invasive IMR to assess microcirculatory function are less than perfect, making early and accurate functional assessment challenging. In the present study, we evaluated CMD in patients with STEMI using the AMR index; therefore, we explored the predictive value of AMR on the occurrence of adverse events in this patient group. The primary findings of the present study are as follows^1^: regarding clinical outcomes, the incidence of MACE was higher in the CMD group than in the non‐CMD group; this was primarily owing to a higher incidence of heart failure readmission^2^; multivariate Cox regression analysis revealed that AMR ≥ 250, age, troponin I detection peak, and BNP were independent risk factors for 1‐year MACE occurrence.

The prognostic effect of CMD occurrence is vital for patients with STEMI, and previous studies have reported that patients with STEMI and an angio‐IMR of >40 U have a significantly higher risk of cardiac‐related death or heart failure admission than controls,^25^ and Scarsini et al.^18^ have reported that patients with STEMI and an invasive IMR of >40 U or CMD assessed by CMR exhibit adverse outcomes mainly caused by heart failure development. However, Fan et al.^11^ have reported that AMR, as a QFR‐derived calculated index, had a good correlation (*r* = .83, *p* < .001) and diagnostic performance (AUC 0.94 [95% CI 0.91–0.97]), with an optimal threshold value of 250 mmHg × s/m for AMR when IMR is ≥25. In the present study, AMR250 was used as the cutoff value owing to the absence of a cutoff value. The analysis revealed a higher incidence of heart failure readmission in patients with CMD; this finding is consistent with that of the abovementioned studies. Nevertheless, this was a retrospective single‐center study, and many prospective studies are warranted to obtain a more accurate cutoff value for AMR in patients with STEMI. Undeniably, PCI significantly decreases in‐hospital mortality in patients with STEMI; however, the incidence of heart failure after STEMI is not rare in clinical settings, with postischemic CMD playing an important role. After reperfusion therapy, CMD is an important pathological change in patients with STEMI and is often associated with poor prognosis.^26^ Therefore, the rapid and accurate assessment of CMD in the acute phase of STEMI may be important.

STEMI is a common acute and critical clinical condition caused by acute myocardial ischemia; it rapidly progresses in the elderly population and is a serious threat to their health. In the present study, baseline data revealed that patients with CMD were significantly older than those without CMD. After performing multifactorial Cox regression analysis, we observed that the age of patients with STEMI is associated with a higher risk of primary endpoint association. Age is a cardiovascular risk factor that cannot be ignored.^27^ To quote a famous line, “You are only as old as your arteries.” Because the population is aging, it is vital to develop new therapeutic approaches to treat serious vascular aging‐related diseases.

BNP is a counter‐regulatory peptide hormone that is primarily synthesized in the ventricular myocardium. As a highly sensitive and specific biomarker for the degree of MI in patients with non‐ST‐elevation acute coronary syndrome, those with high BNP levels at presentation are at a higher risk of death and congestive heart failure^28^; furthermore, high BNP level is an independent predictor of very long‐term all‐cause mortality.^29^ Additional studies have revealed that elevated BNP levels at initial presentation in patients with STEMI are associated with a higher risk of death in the short term.^30^, ^31^ This finding is consistent with that of our multifactorial Cox regression analysis. Therefore, restoring blood flow by opening occluded coronary arteries as early as possible is beneficial to patients with STEMI.

In patients with non‐ST‐elevation acute coronary syndrome who underwent PCI, higher peak preprocedure cardiac troponin I levels were independently associated with 30‐day mortality and composite MACE.^32^ Furthermore, in the 3‐month follow‐up, troponin I was associated with clinical outcomes and cardiac function in patients with STEMI who underwent initial PCI.^33^ However, in the present study, after multifactorial Cox regression analysis, we concluded that the troponin I detection peak in patients with STEMI is associated with a higher risk of the primary endpoint. However, troponin is a marker for myocardial injury. Single‐site measurements of cardiac troponin I in the early stages of STEMI strongly correlate with infarct size.^34^

Because endothelial dysfunction plays a vital role in the cardiovascular complications of diabetes, it is reasonable that there is an association among the history of diabetes, hyperglycemia, and postischemic CMD. A prospective trial of patients without diabetes who presented with STEMI for the first time has confirmed that hyperglycemia on admission is significantly associated with MVO, as defined by CMR.^35^ However, we did not observe this in our study, in which history of diabetes, blood glucose level at admission, and HbA1c were not statistically significant in the CMD and non‐CMD groups. This finding may be related to the lower prevalence of diabetes in the included population and the small sample size.

Compared with invasive physiological assessments, QFR and AMR may be more promptly adopted into the angiography‐based diagnostic and interventional procedural workflow. They do not require the use of specialized guidewires and can be easily repeated multiple times during the procedure. Therefore, QFR and AMR may facilitate the use of physiological assessment in routine procedures in clinical settings. CMD is prevalent in patients with cardiovascular risk factors and is associated with an increased risk of adverse events; therefore, it is an important reason for CAD.^2^ Some researchers have suggested that oxidative stress and inflammatory responses caused by the excessive production and accumulation of cellular reactive oxygen species (ROS) are the key mechanisms that drive CMD development.^36^ In vitro and in vivo studies have reported that increased intracellular ROS concentrations promote the conversion of NO to peroxynitrite radicals, leading to impaired NO‐mediated vasodilation and the increased vasoconstrictor activity of ET‐1 (a vasoconstrictor agonist) by activating the RhoA/Rho‐kinase pathway.^37^, ^38^ However, in the present study, we demonstrated that there is no effective treatment to improve MVO in patients with STEMI; furthermore, there are no large‐scale randomized clinical trials to investigate specific treatment strategies for CMD, the pathophysiological mechanisms underlying CMD in different cardiovascular diseases remain unelucidated, and treatment options for CMD are lacking, warranting additional in‐depth studies to achieve the goal of providing individualized treatment to patients.^2^ Therefore, actively controlling the risk factors such as smoking cessation, rational control of blood pressure and diabetes, lipid management, and treatment of the primary disease are effective approaches to prevent microangiopathy progression and improve angina symptoms. In addition, it is vital to identify high‐risk patients early so as to develop individualized treatment strategies early to improve long‐term prognosis.

Our study still has some limitations, which should be acknowledged. First, this was a retrospective single‐center observational study with a small sample size. Additional prospective multicenter cohort studies are warranted to validate the findings. Second, not all images are suitable for QFR and AMR analyses, possibly resulting in selection bias. Third, individual vessel AMR immediately after primary PCI may not entirely explain the overall patient prognosis, and the focus of our study was limited to analyzing microcirculatory dysfunction in the vascular region of the culprit vessel in patients with STEMI. Therefore, our future studies will focus on the comparative prognosis of UA, NSTEMI, and nonculprit vascular regions with potential microcirculatory dysfunction. Fourth, 1‐year follow‐up MACE was evaluated; our small sample size and short follow‐up period may account for the extremely low number of MIs and revascularizations among the secondary endpoint events; therefore, in our subsequent studies, we will conduct follow‐up for a longer period. In addition, because most of the follow‐ups were conducted via the telephone and returned to the hospital, which was affected by economic conditions and epidemics, no data on coronary re‐examination 1 year after MI are available.

## 5 CONCLUSION

In the present study, we observed that the retrospective use of AMR with a cutoff value of ≥250 after PCI in patients with STEMI can predict a significant difference in the 1‐year MACE rates when compared with a propensity score‐matched group with normal AMR.

## Supporting information

Supporting information.

Supporting information.

Supporting information.

## Data Availability

Data will be made available on request.
