# Supplementary figures and images for: Application of AMR in evaluating microvascular dysfunction after ST‐elevation myocardial infarction
Source: Clin Cardiol. 2023 Nov 24;47(2):e24196. doi: 10.1002/clc.24196 (PMC10823552; doi:10.1002/clc.24196)

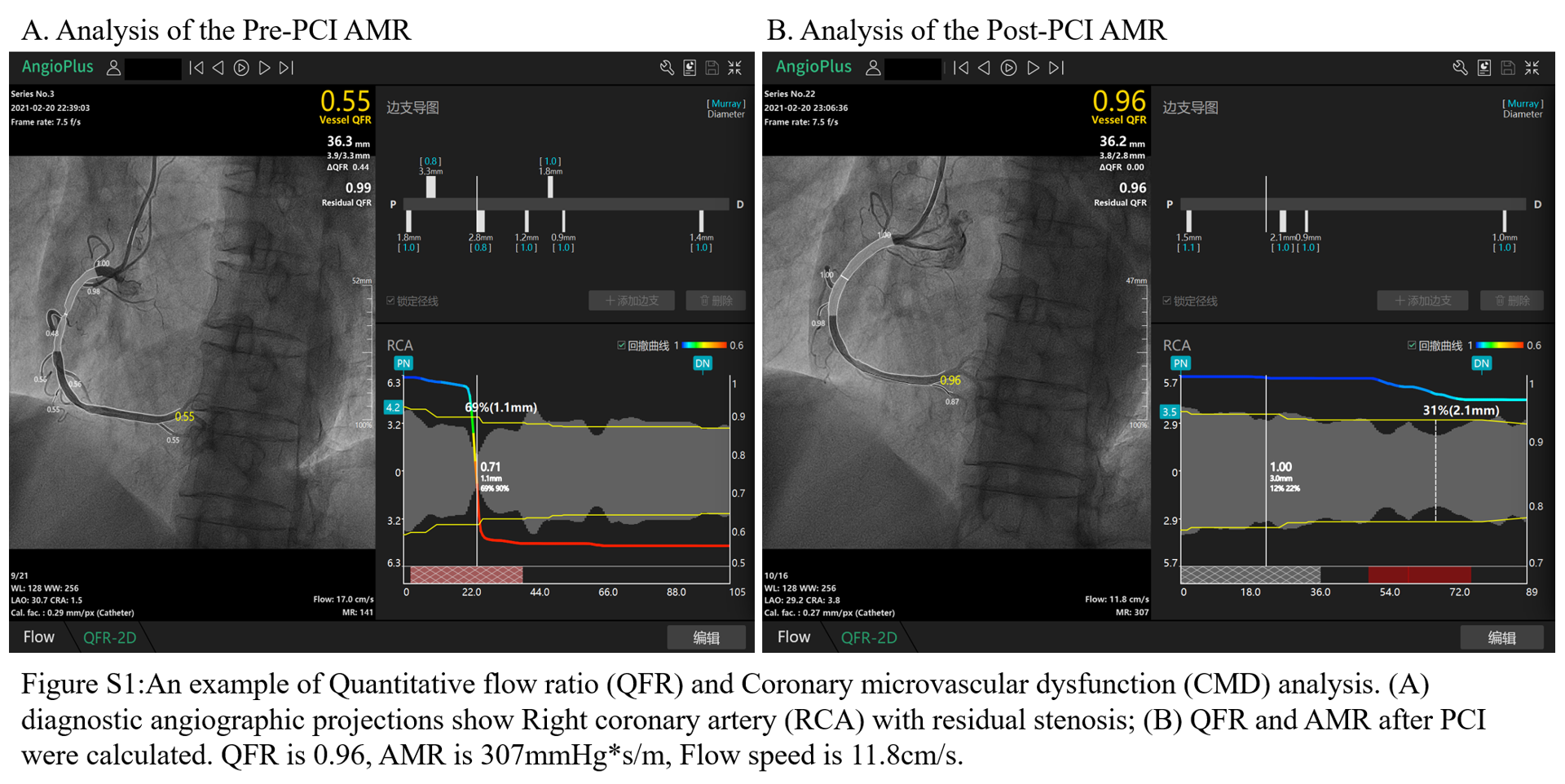

Supplement: Supplementary file 1 — Supporting information. [file CLC-47-e24196-s002.tif]
