# Supplementary material for: Application of AMR in evaluating microvascular dysfunction after ST‐elevation myocardial infarction
Source: Clin Cardiol. 2023 Nov 24;47(2):e24196. doi: 10.1002/clc.24196 (PMC10823552; doi:10.1002/clc.24196)
Supplement: Supplementary file 2 — Supporting information. [file CLC-47-e24196-s001.docx]

Table S1: Baseline characteristics

|  | **Before propensity score matching** | | | | |  | **After propensity score matching** | | | | |
| --- | --- | --- | --- | --- | --- | --- | --- | --- | --- | --- | --- |
|  | **All subjects**  **(n = 506)** | **AMR≥ 250**  **(n = 215)** | **AMR＜250**  **(n = 291)** | **χ²/t/Z** | **P-value** |  | **All subjects**  **(n = 382)** | **AMR≥ 250**  **(n = 191)** | **AMR＜250**  **(n = 191)** | **χ²/t/Z** | **P-**  **value** |
| **Age, y** | 63.00(53.00-73.00) | 67.00(56.00-74.00) | 60.00(51.75-72.00） | -3.827 | 0.000* |  | 65.00(55.00-74.00) | 66.00(55.00-73.00) | 63.00(54.00-74.00) | -0.515 | 0.606 |
| **Male, %** | 416 (82.2) | 167（77.7） | 249（85.6） | 5.267 | 0.022* |  | 308 (80.6) | 153(80.1) | 155(81.2) | 0.067 | 0.796 |
| **Body mass index, kg/m^2^** | 22.64(20.28-24.03) | 22.64(20.28-23.66) | 22.64(20.28-25.35) | -2.391 | 0.017* |  | 22.64(20.28-23.66) | 22.64(20.28-23.66) | 22.64(20.28-23.66) | -0.138 | 0.890 |
| **Cigarette smoking, %** | 229 (45.3) | 90(41.9) | 139(47.8) | 1.741 | 0.187 |  | 172 (45.0) | 86(45.0) | 86(45.0) | 0.000 | 1.000 |
| **Diabetes mellitus, %** | 137 (27.1) | 50(23.3) | 87(29.9) | 2.762 | 0.097 |  | 89 (23.3) | 49(25.7) | 40(20.9) | 1.187 | 0.276 |
| **Hypertension, %** | 265 (52.4) | 109(50.7) | 156(53.6) | 0.420 | 0.517 |  | 197 (51.6) | 100(52.4) | 97(50.8) | 0.094 | 0.759 |
| **Hyperlipidemia, %** | 138 (27.3) | 47(21.9) | 91(31.3) | 5.521 | 0.019* |  | 93(24.3) | 46(24.1) | 47(24.6) | 0.014 | 0.905 |
| **Family history of CAD, %** | 10(2.0) | 4(1.9) | 6(2.1) |  | 1.0 |  | 10(2.6) | 4(2.1) | 6(3.1) |  | 0.751 |
| **Previous MI, %** | 10(2.0) | 4(1.9) | 6(2.1) |  | 1.0 |  | 8(2.1) | 3(1.6) | 5(2.6) |  | 0.724 |
| **Previous CABG, %** | 0(0) | 0(0) | 0(0) |  | - |  | 0(0) | 0(0) | 0(0) |  | - |
| **Previous PCI, %** | 17(3.4) | 4(1.9) | 13(4.5) |  | 0.136 |  | 12(3.1) | 3(1.6) | 9(4.7) |  | 0.140 |
| **Previous stroke, %** | 44(8.7) | 20(9.3) | 24(8.2) | 0.173 | 0.677 |  | 36(9.4) | 18(9.4) | 18(9.4) | 0.000 | 1.000 |
| **Peripheral artery disease, %** | 219(43.3) | 93(43.3) | 126(43.3) | 0.000 | 0.992 |  | 166(43.5) | 83(43.5) | 83(43.5) | 0.000 | 1.000 |
| **Antiplatelet agent, %** | 499(98.6) | 214(99.5) | 285(97.9) |  | 0.247 |  | 377(98.7) | 190(99.5) | 187(97.9) |  | 0.372 |
| **Statin, %** | 496(98.0) | 212(98.6) | 284(97.6) |  | 0.529 |  | 375(98.2) | 188(98.4) | 187(97.9) |  | 1.000 |
| **ACE-inhibitor/ARB, %** | 312(61.7) | 120(55.8) | 192(66.0) | 5.405 | 0.020* |  | 234(61.3) | 109(57.1) | 125(65.4) | 2.824 | 0.093 |
| **LVEF (%)** | 54.00(46.00-59.00) | 54.00(46.00-58.00) | 54.00(46.00-59.00) | -0.726 | 0.468 |  | 54.00(46.00-58.00) | 54.00(47.00-58.00) | 54.00(46.00-58.00) | -0.017 | 0.987 |
| **Fibrinogen, g/L** | 2.97(2.47-3.76) | 2.99(2.49-3.93) | 2.94(2.46-3.70) | -0.804 | 0.422 |  | 2.97(2.49-3.84) | 2.97(2.45-3.84) | 3.03(2.53-3.85) | -0.460 | 0.646 |
| **Albumin, g/L** | 38.58±3.95 | 37.99±3.97 | 39.01±3.90 | 2.913 | 0.004* |  | 38.37±3.86 | 38.30±3.89 | 38.43±3.85 | 0.320 | 0.749 |
| **NEUT*10^9/L** | 7.50(5.57-10.07) | 8.09(5.79-10.46) | 7.16(5.45-9.68) | -2.118 | 0.034* |  | 7.59(5.71-10.17) | 8.11(5.76-10.53) | 7.16(5.65-9.79) | -1.426 | 0.154 |
| **LYM*10^9/L** | 1.47(1.00-2.21) | 1.41(0.95-1.94) | 1.59(1.04-2.37) | -2.250 | 0.024* |  | 1.45(0.98-2.14) | 1.43(1.02-1.97) | 1.47(0.97-2.34) | -0.424 | 0.671 |
| **PLT*10^9/L** | 213.00(174.00-256.25) | 211.00(171.75-254.75) | 215.50(177.75-256.25) | -1.150 | 0.250 |  | 213.00(175.00-256.00) | 211.00(172.00-260.00) | 214.00(178.00-254.00) | -0.707 | 0.480 |
| **WBC*10^9/L** | 10.06（8.13-12.36） | 10.38（8.32-12.45） | 9.68（8.09-12.11） | -1.320 | 0.187 |  | 10.19（8.03-12.44) | 10.44（8.27-12.45) | 9.61（7.87-12.16) | -1.200 | 0.230 |
| **Creatinine, µmol/L** | 80.97(67.56-96.98) | 83.22(65.98-100.99) | 79.25(67.70-94.09) | -1.084 | 0.278 |  | 79.95(66.33-96.73) | 81.90(65.60-99.50) | 78.96(66.40-94.08) | -0.642 | 0.521 |
| **HbA1c%** | 5.90(5.50-6.70) | 5.80(5.50-6.50) | 5.90(5.60-6.80) | -1.650 | 0.099 |  | 5.90(5.50-6.49) | 5.80(5.50-6.60) | 5.90(5.50-6.49) | -0.143 | 0.886 |
| **CRP mg/L** | 29.40(3.88-30.39) | 30.39(4.58-30.39) | 24.36(3.71-30.39) | -1.392 | 0.164 |  | 29.05(3.72-30.39) | 30.39(3.60-30.39) | 27.03(3.77-30.39) | -0.507 | 0.612 |
| **Troponin I, ng/ml** | 7.63(0.89-38.68) | 9.87(0.98-48.20) | 6.38(0.83-34.06) | -1.308 | 0.191 |  | 8.96(0.89-39.99) | 9.21(0.90-48.21) | 8.19(0.83-32.87) | -0.591 | 0.554 |
| **Troponin I detection peak, ng/ml** | 50.00(17.49-50.00) | 50.00(18.07-50.00) | 50.00(16.94-50.00) | -0.100 | 0.920 |  | 50.00(18.57-50.00) | 50.00(20.04-50.00) | 50.00(18.24-50.00) | -0.196 | 0.844 |
| **BNP, pg/ml** | 166.04(59.57-475.48) | 189.19(78.09-485.36) | 142.55(50.00-401.82) | -2.531 | 0.011* |  | 181.57(76.38-481.29) | 181.05(70.91-475.48) | 194.17(81.26-529.13) | -0.586 | 0.558 |
| **Total cholesterol, mmol/L** | 4.55(3.83-5.30) | 4.43(3.74-5.24) | 4.63(3.90-5.33) | -1.757 | 0.079 |  | 4.47(3.76-5.27) | 4.44(3.74-5.30) | 4.52(3.78-5.23) | -0.292 | 0.770 |
| **LDL-c, mmol/L** | 2.78(2.25-3.40) | 2.67(2.14-3.31) | 2.85(2.36-3.44) | -2.297 | 0.022* |  | 2.69(2.15-3.35) | 2.70(2.10-3.40) | 2.69(2.20-3.35) | -0.355 | 0.723 |
| **Glucose, mmol/L** | 6.21(5.33-8.05) | 6.11(5.27-7.62) | 6.28(5.37-8.16) | -0.591 | 0.555 |  | 6.15(5.30-7.92) | 6.18(5.33-8.51) | 6.15(5.29-7.65) | -0.878 | 0.380 |

Values are presented as the mean ± standard deviation, median (25th percentile, 75th percentile) or n (%).

AMR, angio-derived microcirculatory resistance; CAD, coronary artery disease; CABG, coronary artery bypass graft; PCI, percutaneous coronary intervention; MI, myocardial infarction; ACE-inhibitor, angiotensin-converting-enzyme inhibitor; ARB, angiotensin II receptor blocker. LVEF, left ventricular ejection fraction; NEUT, Neutrophil count; LYM, Lymphocyte count; PLT, blood platelet count; WBC, white blood cell count; HbA1c, glycated hemoglobin A1c; CRP, C-reactive protein; BNP, brain natriuretic peptide; LDL-c =low density lipoprotein cholesterol; Glucose, First admission blood glucose.
