# Supplementary material for: Application of AMR in evaluating microvascular dysfunction after ST‐elevation myocardial infarction
Source: Clin Cardiol. 2023 Nov 24;47(2):e24196. doi: 10.1002/clc.24196 (PMC10823552; doi:10.1002/clc.24196)
Supplement: Supplementary file 3 — Supporting information. [file CLC-47-e24196-s003.docx]

Table S2: Coronary angiography characteristics

|  | **Before propensity score matching** | | | | |  | **After propensity score matching** | | | | |
| --- | --- | --- | --- | --- | --- | --- | --- | --- | --- | --- | --- |
|  | **All subjects**  **(n = 506)** | **AMR≥ 250**  **(n = 215)** | **AMR＜250**  **(n = 291)** | **χ²** | **P-value** |  | **All subjects**  **(n = 382)** | **AMR≥ 250**  **(n = 191)** | **AMR＜250**  **(n = 191)** | **χ²** | **P-**  **value** |
| **Medications in operation Bivalirudin, %** | 81(16.0) | 37(17.2) | 44(15.1) | 0.401 | 0.526 |  | 64(16.8) | 33(17.3) | 31(16.2) | 0.075 | 0.784 |
| **thrombus aspiration, %** | 102(20.2) | 47(21.9) | 55(18.9) | 0.673 | 0.412 |  | 72(18.8) | 42(22.0) | 30(15.7) | 2.465 | 0.116 |
| **Radial artery approach, %** | 495(97.8) | 212(98.6) | 283(97.3) |  | 0.368 |  | 375(98.2) | 190(99.5) | 185(96.9) |  | 0.122 |
| **Multivessel disease, %** | 213(42.1) | 87(40.5) | 126(43.3) | 0.407 | 0.523 |  | 170(44.5) | 78(40.8) | 92(48.2) | 2.077 | 0.149 |
| **One vessel disease, %** | 169(33.4) | 77(35.8) | 92(31.6) | 0.980 | 0.322 |  | 123(32.2) | 69(36.1) | 54(28.3) | 2.698 | 0.100 |
| **Two vessel disease, %** | 150(29.6) | 63(29.3) | 87(29.9) | 0.021 | 0.885 |  | 111(29.1) | 54(28.3) | 57(29.8) | 0.114 | 0.735 |
| **Three vessel disease, %** | 179(35.4) | 73(34) | 106(36.4) | 0.331 | 0.565 |  | 143(37.4) | 68(35.6) | 75(39.3) | 0.548 | 0.459 |
| **Left main disease, %** | 8(1.6) | 2(0.9) | 6(2.1) |  | 0.477 |  | 5(1.3) | 0(0) | 5(2.6) |  | 0.061 |
| **Culprit vessel, n** | Total (n = 553) | n=233 | n=320 |  |  |  | Total (n = 415) | n=205 | n=210 |  |  |
| **Left anterior descending artery, %** | 289(52.3) | 111 (47.6) | 178 (55.6) | 3.446 | 0.063 |  | 217 (52.3) | 101 (49.3) | 116 (55.2) | 1.482 | 0.223 |
| **Left circumflex artery, %** | 88(15.9) | 45 (19.3) | 43 (13.4) | 3.479 | 0.062 |  | 65(15.7) | 39 (19.0) | 26 (12.4) | 3.466 | 0.063 |
| **Right coronary artery, %** | 172(31.1) | 75 (32.2) | 97 (30.3) | 0.222 | 0.638 |  | 132(31.8) | 65 (31.7) | 67 (31.9) | 0.002 | 0.966 |
| **Left main disease, %** | 4(0.8) | 2(0.9) | 2(0.7) |  | 1.000 |  | 1(0.2) | 0(0) | 1(0.5) |  | 1.000 |
| **Balloon pre-dilation, %** | 495(97.8) | 208(96.7) | 287(98.6) |  | 0.217 |  | 372(97.4) | 184 (96.3) | 188 (98.4) |  | 0.337 |
| **Stents per vessel** | 1.55±0.80 | 1.58±0.75 | 1.53±0.83 | -0.616 | 0.538 |  | 1.55±0.78 | 1.58±0.75 | 1.52±0.81 | -1.099 | 0.272 |
| **Balloon post-dilation, %** | 341(67.4) | 136(63.3) | 205(70.4) | 2.909 | 0.088 |  | 262(68.6) | 125(65.4) | 137(71.7) | 1.750 | 0.186 |
| **Drug-eluting stents placed, %** | 498(98.4) | 213(99.1) | 285(97.9) |  | 0.477 |  | 377(98.7) | 189(99.0) | 188(98.4) |  | 1.000 |
| **Drug-coated balloon angioplasty, %** | 4(0.8) | 0(0) | 4(1.4) |  | 0.140 |  | 3(0.8) | 0(0) | 3(1.6) |  | 0.248 |
| **Non-drug-coated balloon angioplasty, %** | 4(0.8) | 2(0.9) | 2(0.7) |  | 1.000 |  | 3(0.8) | 2(1.0) | 1(0.5) |  | 1.000 |
| **post-PCI TIMI flow grade, %**  **0** | 0(0) | 0(0) | 0(0) |  |  |  | 0(0) | 0(0) | 0(0) |  |  |
| **1** | 1(0.2) | 1(0.5) | 0(0) |  | 0.425 |  | 1(0.3) | 1(0.5) | 0(0) |  | 1.000 |
| **2** | 8(1.6) | 8(3.7) | 0(0) |  | 0.001^*^ |  | 6(1.6) | 6(3.1) | 0(0) |  | 0.030^*^ |
| **3** | 497(98.2) | 206(95.8) | 291(100) |  | 0.000^*^ |  | 375(98.2) | 184(96.3) | 191(100) |  | 0.015^*^ |

Values are presented as the mean ± standard deviation, median (25th percentile, 75th percentile) or n (%).

AMR, angio-derived microcirculatory resistance; TIMI, thrombolysis in myocardial infarction.
